# Profound Thrombocytopenia and Dyspnea 11 Days After Cardiac Surgery

**DOI:** 10.1002/ajh.27691

**Published:** 2025-05-01

**Authors:** Sebastian Vuong, Menaka Pai, Sarah Patterson, Theodore E. Warkentin

**Affiliations:** ^1^ Department of Pathology and Molecular Medicine McMaster University Hamilton Ontario Canada; ^2^ Department of Medicine McMaster University Hamilton Ontario Canada; ^3^ Service of Benign Hematology Hamilton Health Sciences, Hamilton General Hospital Hamilton Ontario Canada; ^4^ Transfusion Medicine Hamilton Regional Laboratory Medicine Program Hamilton Ontario Canada

**Keywords:** disseminated intravascular coagulation (DIC), hemorrhage, heparin, posttransfusion purpura (PTP), thrombocytopenia

## 1 Case Presentation

**A 59‐year‐old Caucasian female with hypertension and dyslipidemia presented to the hospital with dyspnea. Her platelet count was 7 × 10** ^**9**^ **/L (reference range [RR], 150–400). Eleven days earlier, she had undergone an elective valve‐sparing Bentall procedure (replacement of hemi‐aortic arch) and ascending aorta repair with cardiopulmonary bypass (CPB) for aortic root aneurysm. Her postoperative course was uneventful, with discharge on postoperative day (POD) 4. The patient reported no bleeding symptoms/signs, and petechiae were not present**.

Platelet count declines are universal post‐cardiac surgery, with platelet count recovery to preoperative baseline expected by the seventh POD, and continued platelet count increase that typically peaks by POD14 [1]; thus, a platelet count of 7 × 10^9^/L on POD11 is highly abnormal and sufficiently reduced to be classified as “profound” thrombocytopenia (< 20 × 10^9^/L) [2]. Yet, this patient presented with dyspnea, not bleeding. The urgent task was to identify promptly the cause of her dyspnea and to address the profound thrombocytopenia with the key question: did a single diagnosis explain both?

**Vital signs were BP 98/59, HR 102/min, RR 23/min, temperature 38.5°C; oxygen saturation was 95% (room air). Urgent echocardiography showed normal ventricular contractility without valve abnormalities; however, a moderate‐to‐large pericardial effusion was present; subtle right ventricular diastolic collapse suggested partial or incipient cardiac tamponade. The cardiologists were concerned that the pericardial fluid was blood rather than serous fluid, that is hemorrhagic pericarditis. However, risk of pericardiocentesis was felt to be extremely high due to thrombocytopenia; thus, urgent hematology consultation was requested**.

There is a limited differential diagnosis for profound thrombocytopenia, particularly given the normal platelet count just 11 days earlier. The differential diagnosis includes: pseudothrombocytopenia (spurious thrombocytopenia); consumptive thrombocytopenia (disseminated intravascular coagulation [DIC] secondary to infection/shock, thrombotic microangiopathy [TMA], or immune heparin‐induced thrombocytopenia [HIT]); or destructive thrombocytopenia (antibody‐mediated platelet clearance by drug‐dependent antibodies, autoantibodies, or alloantibodies). A reasonable first diagnostic step is to evaluate the complete blood count (CBC), along with peripheral blood film review.

**The hemoglobin measured 7.7 g/dL (RR, 13.0–18.0) and the white blood cell (WBC) count was 14.0 × 10** ^**9**^ **/L (RR, 4.0–11.0). Repeat CBC confirmed profound thrombocytopenia (8 × 10** ^**9**^ **/L). The mean platelet volume (MPV)—at 12.2 fL (RR, 9.3–12.5)—was higher than the preoperative value (9.8 fL). The automated WBC differential showed (absolute count values ×10** ^**9**^ **/L): neutrophils, 10.6 (RR, 2.0–7.5); lymphocytes, 2.3 (RR, 1.5–4.0); monocytes, 0.8 (RR, 0.2–0.8); eosinophils, 0 (RR, 0–0.4); and basophils, 0 (RR, 0–0.1); nucleated red blood cells (nRBCs) were quantitated at 0.2 × 10** ^**9**^ **/L (RR, undetectable). Manual blood film review confirmed the absence of platelets. No “toxic” WBCs (toxic granulation or vacuolization) were evident. Polychromatophilic red cells were seen, but no red cell fragments (schistocytes) were observed**.

Absence of large platelet aggregates and platelets aggregated around neutrophils (“satellitism”) ruled out “pseudothrombocytopenia” (spurious thrombocytopenia) [3]. Besides profound thrombocytopenia, the patient had leukocytosis (neutrophilia), anemia, polychromasia, and normoblastemia. One report of post‐cardiac surgery thrombotic thrombocytopenic purpura (TTP) provided evidence that preexisting quiescent anti‐ADAMTS13 autoantibodies can become clinically relevant during the proinflammatory milieu that inevitably follows cardiac surgery, though absence of schistocytes argued against TTP in this patient [4, 5]. The next step was to compare the CBC with the recent post‐surgery CBCs.

**FIGURE 1:**
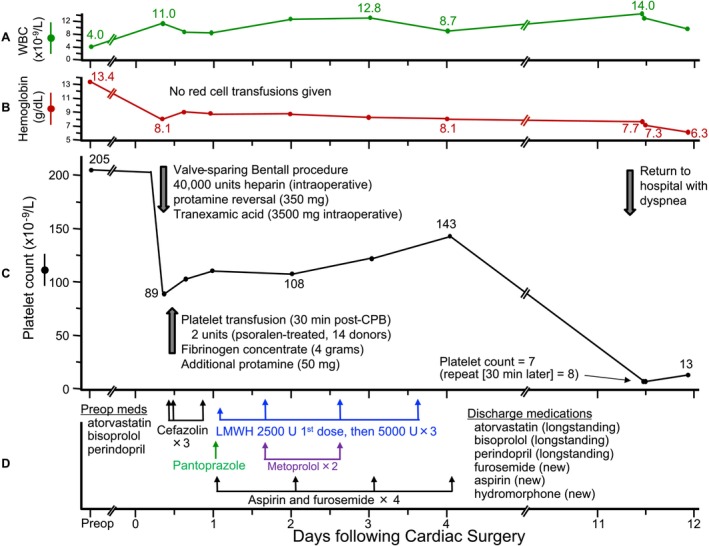
Serial blood counts, clinical events, medications, and blood products in relation to cardiac surgery and at re‐presentation to hospital on POD11. (A) White blood cell values. (B) Hemoglobin values. (C) Platelet count values (including timing of perioperative platelet transfusions). (D)Medications administered, including intraoperative unfractionated heparin and postoperative low‐molecular‐weight heparin (dalteparin). CPB, cardiopulmonary bypass; U, units; WBC, white blood cell. [Color figure can be viewed at wileyonlinelibrary.com]

**Results of the patient's pre‐ and postoperative serial CBCs are shown in Figure** **1** **A–C. The preoperative hemoglobin was 13.4 g/dL and fell to 8.1 g/dL (nadir) on POD4. No red cell concentrates (RCCs) were given. The preoperative WBC count was 4.0 × 10** ^**9**^ **/L which increased to 12.8 × 10** ^**9**^ **/L (peak) on POD3. The normal preoperative platelet count (205 × 10** ^**9**^ **/L) fell to 89 × 10** ^**9**^ **/L immediately post‐surgery. A review of the anesthetic record confirmed usual heparin anticoagulation during CPB (40 000 units), usual protamine reversal (400 mg), and unremarkable CPB time (139 min). The patient received intraoperative blood products: platelet transfusions (2 × 250 mL pooled psoralen‐treated platelets, blood group A matched to the patient's blood group A status) and 4 g of fibrinogen concentrate (due to subjective “oozing” per surgical team); 2 days later her platelet count was 108 × 10** ^**9**^ **/L, and by POD4 had risen to 143 × 10** ^**9**^ **/L. Her postoperative course was unremarkable, with discharge to home on POD4**.

Her hemoglobin fell to 8.1 g/dL**—**a common consequence of hemodilution and surgical bleeding after cardiac surgery. It is common practice not to transfuse RCCs in the absence of marked bleeding or cardiovascular dysfunction unless the hemoglobin falls to < 8.0 g/dL [6], and she indeed received no RCCs intra‐ or postoperatively. The further decline in hemoglobin to 7.7 g/dL (repeat, 7.3 g/dL) on POD11 was worrisome and consistent with hemorrhagic pericarditis secondary to bleeding into the pericardium. Her postoperative platelet count trend was in keeping with usual post‐cardiac surgery changes, and for a patient whose POD4 platelet count was 143 × 10^9^/L, the expectation by POD11 would be for the platelet count to have rebounded and exceed at least 400 × 10^9^/L [7].

Her leukocytosis/neutrophilia was consistent with either infection or physiological “stress” (dyspnea, anemia). Given the normoblastemia, the hematologist considered a consumptive thrombocytopenia including DIC states or the coagulation‐activating adverse drug reaction, HIT, within the differential diagnosis. This is because normoblastemia is common in conditions of bone marrow stress including lactic acidemia with DIC [8] and severe, symptomatic anemia [9]. The hematologist ordered markers of hypoperfusion (serum lactate, liver enzymes as markers of acute ischemic hepatitis [“shock liver”]) and also screened for DIC (prothrombin time [PT], activated partial thromboplastin time [APTT], fibrinogen, d‐dimer). Testing for HIT antibodies was requested.

**Chemistry showed normal lactate 1.9 mmol/L (RR, 0.7–2.1), but elevated ALT 148 U/L (RR, 0–34) and AST 81 U/L (RR, 18–34); the LDH was also elevated at 338 U/L (RR, 120–250). The PT was 14.3 s (RR, 9.4–12.5) with a corresponding international normalized ratio (INR) of 1.3 (RR, 0.8–1.1); the APTT measured 24 s (RR, 25–37), fibrinogen 670 mg/L (RR, 200–390), and the d‐dimer (HemosIL d‐Dimer HS 500 assay performed on ACL TOP Family 50 series instrument; Werfen, Bedford, MA, USA) measured 18 630 μg/L FEU (RR, < 500). Blood cultures were drawn. Rapid HIT testing was not available at this hospital, thus screening PF4/polyanion enzyme‐linked immunosorbent assay (ELISA) results were pending. Since the patient presented on Friday afternoon, these HIT antibody test results were not expected until Monday afternoon**.

Although the normal lactate argued against an overt “shock” state, the moderately elevated hepatic transaminases suggested that hemorrhagic pericarditis could have resulted in cardiovascular dysfunction and associated hepatic congestion. The coagulation testing suggested the possibility of DIC per International Society on Thrombosis and Haemostasis (ISTH) DIC criteria: platelet count < 50 × 10^9^/L (2 points) and greatly elevated d‐dimer levels > 10 000 μg/mL FEU (3 points) totaling 5 points suggesting possible DIC [10]. However, the PT and corresponding INR values were only minimally elevated, and the fibrinogen was greatly elevated, as expected in the proinflammatory postcardiac surgery context [11]. Although the ISTH DIC score was possibly consistent with DIC, the concern was that the profound thrombocytopenia seemed more in keeping with a destructive (rather than consumptive) thrombocytopenia and, importantly, that the greatly elevated d‐dimer could reflect resorption of blood (and fibrin) from the large (presumed) hemorrhagic pericardial effusion. Moreover, the complete absence of red cell fragments also argued against a diagnosis of severe DIC.

The hematologist calculated a low pretest probability for HIT per the 4Ts score [12]: (a) *T*hrombocytopenia = 0 points (platelet count < 20 × 10^9^/L); (b) *T*iming of onset of thrombocytopenia = 2 points (onset between 5 and 10 days); (c) *T*hrombosis = 0 (no thrombosis); (d) o*T*her explanation for thrombocytopenia = 1 point (strong suspicion for destructive thrombocytopenia). Given the low 4Ts score (3 points) [12]—and concern of hemorrhagic pericarditis—the decision was made to hold off on anticoagulation therapy.

To evaluate further explanations for destructive thrombocytopenia such as drug‐induced immune thrombocytopenia (D‐ITP), the hematologist focused on the recent initiation of new medications [13] as well as the intraoperative platelet transfusions, which raised the possibility of posttransfusion purpura (PTP) triggered by the receipt of platelet alloantigen‐containing blood products in a patient previously sensitized through pregnancy or prior transfusion. Alloantibodies to human platelet antigen (HPA)—with concomitant reactivity against autologous platelet antigens—cause a profound destructive thrombocytopenia within 5–10 days of transfusion. Accordingly, the EMR and patient were queried for drugs implicated in D‐ITP, and further history was sought regarding previous pregnancies or remote transfusions.

**The patient had received cefazolin 2 g IV × 2 doses intraoperatively, plus a further 2 g IV dose 8 h post‐surgery (Figure** **1D** **); no further cefazolin or any other antibiotic was subsequently given. Other new medications included: subcutaneous dalteparin (2500 U first dose; then 5000 U daily until POD4); pantoprazole (40 mg IV given once on POD1), metoprolol (PODs 1 through 3), furosemide (40 mg po daily), and aspirin (81 mg enteric‐coated po daily); furosemide and aspirin were continued on discharge. Atorvastatin, bisoprolol, perindopril—all longstanding medications—were resumed on discharge. She had two prior uncomplicated pregnancies, and there was no remote history of blood transfusions. The hematologist referred blood samples to national reference laboratories to perform HPA antibody testing and HPA genotyping; however, results were not expected to be immediately forthcoming**.

Although cefazolin is a documented cause of D‐ITP [14], she had not received this antibiotic for over 10 days. The antibiotic cefotetan can cause delayed‐onset severe immune hemolysis (a potentially fatal complication that led to cefotetan discontinuation) [15], however, delayed presentation of D‐ITP from a drug administered more than a week earlier is not well‐established. In contrast, delayed presentations of HIT (“autoimmune HIT”) can explain thrombocytopenia and thrombosis presenting after discharge from heart surgery [16, 17] though the overall picture seemed low probability for HIT. The patient's longstanding medications (atorvastatin, bisoprolol, perindopril) are not listed as explanations for D‐ITP in review articles [13, 14], and in any event are unlikely culprits given her long‐term uneventful exposure. Aspirin and furosemide—newly started post‐surgery—are possible triggers of D‐ITP [14, 18] and were thus discontinued as a precautionary measure. Aspirin cessation was especially appropriate given profound thrombocytopenia and possible hemorrhagic pericarditis.

The history of previous pregnancies and intraoperative platelet transfusions made PTP a plausible diagnosis; indeed, approximately one‐fifth present post‐cardiovascular surgery [19]. PTP represents an anamnestic reaction usually involving the HPA‐1a/b alloantigen system, whereby an HPA‐1b/1b individual (2% of the population) forms high‐titer platelet‐reactive anti‐HPA‐1a alloantibodies following the receipt of blood product (RCCs, platelets) containing HPA‐1a alloantigen [20]. Through unclear mechanisms, the patient's own HPA‐1b/1b platelets are destroyed during the intense anamnestic anti‐HPA‐1a immune response. Thrombocytopenia is usually profound with a mortality of ~10% due to bleeding [19].

At this point, the hematologist's differential diagnosis focused on three entities: PTP, D‐ITP (aspirin or furosemide), or severe aHIT. Fortunately, treatment with high‐dose intravenous immunoglobulin (IVIG) usually interrupts platelet destruction by reticuloendothelial system macrophages (PTP, D‐ITP) [13, 14, 20] and by HIT antibody‐mediated platelet activation [21]—thus IVIG was prescribed.

**FIGURE 2:**
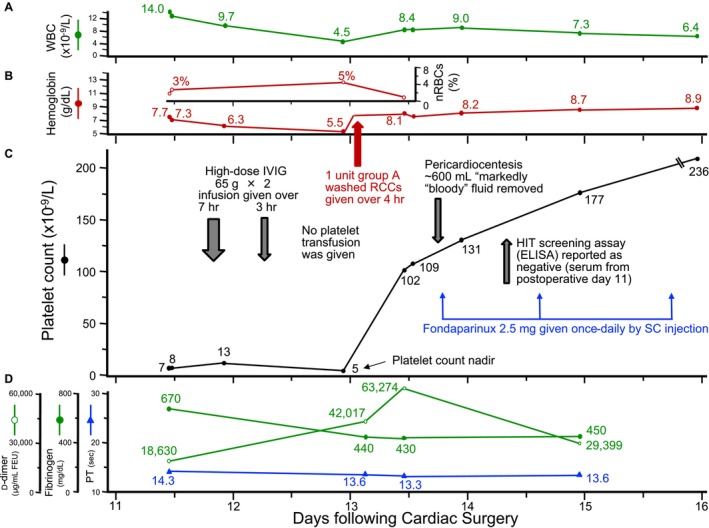
Serial blood counts, clinical events, blood transfusions, and selected medications given following re‐presentation to hospital. (A) White blood cell values. (B) Hemoglobin and nucleated red blood cell (nRBC) values. (C) Platelet count values. (D) Coagulation tests (d‐dimer, fibrinogen, prothrombin time). ELISA, enzyme‐linked immunosorbent assay; FEU, fibrinogen equivalent unit; hr, hours; IVIG, intravenous immunoglobulin; nRBCs, nucleated red blood cells; PT, prothrombin time; RCCs, red cell concentrates; SC, subcutaneous; U, units; WBC, white blood cell. [Color figure can be viewed at wileyonlinelibrary.com]

**The patient received high‐dose IVIG (IVIGnex; Immune Globulin Intravenous [Human], 10%, Grifols, Mississauga, ON, Canada) 65 g with an additional 65 g given the following day (adjusted dosing per patient height 1.71 m and weight, 75 kg). There was no immediate improvement in the platelet count (Figure** **2** **). Fourteen hours post‐infusion, the platelet count was only 5 × 10** ^**9**^ **/L and the d‐dimer level rose to 42 017 μg/mL FEU; however, a marked platelet count increase to 102 × 10** ^**9**^ **/L occurred just 13 h later (with further d‐dimer rise to 63 274 μg/mL FEU). The hemoglobin also decreased following administration of IVIG, from 7.3 to 6.3 mg/dL, and the next day to 5.5 mg/dL. One unit of Group A washed RCC was transfused and the post‐transfusion hemoglobin increased to 8.1 g/dL. The patient underwent drainage of the pericardial fluid with 600 mL of “markedly bloody” fluid removed with subsequent resolution of dyspnea. Two hours post‐pericardiocentesis, low‐dose fondaparinux thromboprophylaxis (2.5 mg/day) was started**.

The dramatic increase in platelet count was consistent with one of the three disorders under consideration (PTP, D‐ITP, aHIT). Fondaparinux thromboprophylaxis was commenced once the profound thrombocytopenia resolved, given residual uncertainty regarding aHIT. In this situation, local availability of a rapid PF4/polyanion immunoassay would have been diagnostically helpful [22]. For example, a negative PF4/polyanion chemiluminescence immunoassay (CLIA) in a low‐probability context would have essentially ruled out HIT (post‐test probability < 1%), although a positive test would have required further investigation for platelet‐activating antibodies [22]. The hemoglobin decrease could be explained either by further bleeding or IVIG‐associated alloimmune hemolysis (passive anti‐A alloantibodies hemolyzing recipient group A red cells) [23].

**A negative test for HIT antibodies (polyspecific PF4/polyanion ELISA) was received Monday afternoon. Testing for alloimmune hemolysis was not performed**.

The negative ELISA essentially ruled out HIT [22]. Investigations for PTP involve [20] (a) typing the patient's platelets to demonstrate HPA‐1b/1b status and (b) demonstrating high‐titer anti‐HPA‐1a alloantibodies. Results are usually not available for several days.

**Aspirin and furosemide were resumed on POD16 and POD18, respectively, without thrombocytopenia recurrence. She was discharged home on POD20 (platelet count 315 × 10** ^**9**^ **/L; hemoglobin 8.5 g/dL). The reference laboratory reported on POD18 that the patient was homozygous HPA‐1bb (polymerase chain reaction with sequence‐specific primers); subsequently, high‐titer anti‐HPA‐1a alloantibodies were identified by glycoprotein‐dependent ELISA and by platelet antibody bead array with the Luminex platform (PAK Lx, Werfen) (reported POD21)**.

## 2 Discussion

This patient had a definitive diagnosis of PTP, which presented as dyspnea (secondary to hemorrhagic pericarditis) and profound thrombocytopenia that became apparent 11 days after intraoperative platelet transfusion for a female patient (with two remote pregnancies) for a Bentall procedure requiring CPB. Several issues warrant further discussion.

First, the clinical picture required diagnostic consideration of severe aHIT. Although there was no further exposure to heparin after POD4, aHIT can explain thrombocytopenia beginning after hospital discharge [16, 17]. Dyspnea in such a patient could represent thrombosis (pulmonary embolism). Moreover, a markedly elevated d‐dimer level is a typical feature in consumptive thrombocytopenic disorders including severe aHIT. However, in this case, the d‐dimer elevation likely reflected the large hemorrhagic pericardial effusion with ongoing resorption/lysis of fibrin clots. This case illustrates the importance of interpreting laboratory values in the appropriate clinical context. Ultimately, the negative PF4/polyanion‐ELISA ruled out HIT.

Second, the case highlights the challenge faced by clinicians when crucial specialized laboratory test results are not available for a few days (e.g., HIT antibody tests) or a few weeks (e.g., platelet alloantigen typing). For this patient, having rapid HIT testing available would have been enormously valuable, as a negative result would have shifted hematologist attention away from aHIT and toward the two most plausible destructive thrombocytopenias, D‐ITP and PTP.

Third, PTP is an ultrarare adverse effect of transfusion with an even lower frequency after introduction of universal RCC leukodepletion (which occurred in Canada in the late 1990s) [24]. However, this case occurred in April 2024, approximately 6 months after the introduction of psoralen‐treated platelets in Canada. Although this likely was coincidental, ongoing surveillance for adverse transfusion reactions—as occurs in the province of Ontario through “Transfusion‐Transmitted Injury Surveillance System (TTISS)” [23, 25]–could provide a signal if a notable increase in PTP case recognition occurs, perhaps due to changes in platelet product manufacturing.

## 3 Summary

We report a patient who developed dyspnea, anemia, and profound thrombocytopenia 11 days following cardiac surgery. The ultimate explanation was PTP resulting in hemorrhagic pericarditis. A confusing aspect of the case was the dramatically increased d‐dimer value that was ironically likely related to the hemorrhagic pericardial effusion. This case underscores the importance of considering a broad differential diagnosis for postoperative thrombocytopenia, and to consider carefully the most plausible explanation, given that various explanations have different management strategies which often lie in opposition to one another.

## Consent

The patient provided written permission for this case report (institutional approval is not required for case reports in our medical community).

## Conflicts of Interest

Theodore E. Warkentin has provided consulting services to Instrumentation Laboratory (Werfen), Paradigm Pharmaceuticals, and Veralox; and has provided expert witness testimony relating to HIT and non‐HIT thrombocytopenia. Menaka Pai has received lecture honoraria from Pfizer; has provided consulting services to Hemostasis Reference Laboratory (Universal Biosensors); and has provided expert witness testimony related to non‐HIT thrombocytopenia. The other authors declare no conflicts of interest.

## Data Availability

All data generated or analyzed during this study are included in this published article.
